# Decreased risk adjusted 30-day mortality for hospital admitted injuries: a multi-centre longitudinal study

**DOI:** 10.1186/s13049-018-0485-2

**Published:** 2018-04-03

**Authors:** Robert Larsen, Denise Bäckström, Mats Fredrikson, Ingrid Steinvall, Rolf Gedeborg, Folke Sjoberg

**Affiliations:** 1 1Department of Clinical and Experimental Medicine, Linkoping University, Linkoping, Sweden; 2 Department of Anaesthesiology and Intensive Care, University Hospital Linkoping, Linkoping University, S-58185 Linkoping, Sweden; 3 Department of Medical and Health Sciences, Linkoping University, Norrkoping, Sweden; 4 Department of Anaesthesiology and Intensive Care, Linkoping University, Norrkoping, Sweden; 5 Department of Medical and Health Sciences, Linkoping University, Norrkoping, Sweden; 6 Department of Hand Surgery, Plastic Surgery and Burns, Linkoping University, Linkoping, Sweden; 7 Department of Surgical Sciences, Anaesthesiology and Intensive Care, Uppsala University, Uppsala, Sweden

**Keywords:** Risk-adjusted mortality, ICISS, Trauma, Injury, Nationwide, Epidemiological

## Abstract

**Background:**

The interpretation of changes in injury-related mortality over time requires an understanding of changes in the incidence of the various types of injury, and adjustment for their severity. Our aim was to investigate changes over time in incidence of hospital admission for injuries caused by falls, traffic incidents, or assaults, and to assess the risk-adjusted short-term mortality for these patients.

**Methods:**

All patients admitted to hospital with injuries caused by falls, traffic incidents, or assaults during the years 2001–11 in Sweden were identified from the nationwide population-based Patient Registry. The trend in mortality over time for each cause of injury was adjusted for age, sex, comorbidity and severity of injury as classified from the International Classification of diseases, version 10 Injury Severity Score (ICISS).

**Results:**

Both the incidence of fall (689 to 636/100000 inhabitants: *p* = 0.047, coefficient − 4.71) and traffic related injuries (169 to 123/100000 inhabitants: *p* < 0.0001, coefficient − 5.37) decreased over time while incidence of assault related injuries remained essentially unchanged during the study period. There was an overall decrease in risk-adjusted 30-day mortality in all three groups (OR 1.00; CI95% 0.99–1.00). Decreases in traffic (OR 0.95; 95% CI 0.93 to 0.97) and assault (OR 0.93; 95% CI 0.87 to 0.99) related injuries was significant whereas falls were not during this 11-year period.

**Discussion:**

Risk-adjustment is a good way to use big materials to find epidemiological changes. However after adjusting for age, year, sex and risk we find that a possible factor is left in the pre- and/or in-hospital care.

**Conclusions:**

The decrease in risk-adjusted mortality may suggest changes over time in pre- and/or in-hospital care. A non-significantdecrease in risk-adjusted mortality was registered for falls, which may indicate that low-energy trauma has not benefited for the increased survivability as much as high-energy trauma, ie traffic- and assault related injuries.

## Background

Substantial efforts have been made for decades to improve injury prevention and implement systematic improvements of trauma care [1], but it remains a challenge to measure the impacts of these efforts in terms of changes in injury incidence and mortality. It has been reported that traffic-related mortality has decreased over time, but no such improvement has been described for fall-related injuries [2]. In order to try to understand the potential impact of health care interventions on injury mortality it is essential to adjust for changes in injury incidence, injury severity, and patient baseline characteristics such as comorbidity.

For this study we elected to use the national patient registry, instead of a specialised trauma registry. This allows a nation-wide population-based approach and avoids some concerns regarding coverage and selection bias inherent for trauma registries. For all types of data sources concerns regarding the validity of data must be addressed. The International Classification of disease Injury Severity Score (ICISS) has been developed for use with large administrative registries [3, 4]. Using ICISS for risk adjustment accurately predicts the chance of survival, and is comparable to the Injury Severity Score (ISS) in trauma [5].

This study focuses on three causes of injury. Traffic injury is the dominant type of high-energy trauma causing hospital admission and has a substantial mortality rate. This injury type is therefore expected to be well suited to reflect the potential effects of an improved trauma care system, since these efforts have mainly focused on high-energy trauma. While falls are the most common cause of injury resulting in hospital admission, they are dominated by low-energy trauma and are usually not targeted by the trauma care system. Falls, however, still have a large impact on public health and health-care consumption [6]. Injuries resulting from assault are the third category in focus for this study. Violence is one of the most common causes of death among young males worldwide [7]. Hospitalised victims of violent crimes, however, have lower mortality compared to traffic injury [8] and incidence can be expected to depend on other factors than those underlying traffic injury incidence.

The aim of the present study was to estimate changes in incidence and risk-adjusted mortality over time among patients in Sweden who were admitted to hospital with injuries caused by falls, traffic incidents, and assaults.

## Methods

### Patients studied

All hospital admissions for trauma caused by fall, traffic incident, or assault during the years 2001 to 2011 in Sweden were retrieved from the National Patient Registry. Patients who died before reaching hospital or who had injuries that did not require hospital admission were not included in the study population. For patients who were transferred between departments during treatment for the same injury we used the first record in the registry as the date of admission and diagnoses, and the last date of that admission for the date of discharge. All records with an International Classification of Diseases version 10 (ICD-10) [9] main diagnosis in the range of S00-T80 (trauma diagnoses), excluding T78 (adverse effects), or with an external cause of injury in the range V01-Y98.9, were selected during the first step. These records were linked to all records in the Cause of Death Registry that had “injury” as the main cause of death (V01-Y98.9). Records with missing information on age, sex, date of admission, or mechanism of injury were excluded from the analyses.

**Fig. 1:**
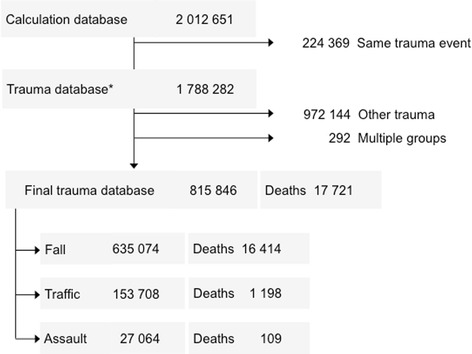
Flow chart showing the selection of the patients studied. “Other trauma” includes all other injuries. “Multiple groups” are the observations that were coded in two groups at the same time. *The Diagnosis-specific Survival Probability was calculated on the Trauma database

Records in which the cause of injury was “fall” (W00-W19), “traffic incident” (V01-V99), or “assault” (X85-Y09) were then selected for further study (Fig. 1). A few observations (*n* = 292, 0.036%) were classified in more than one of the groups, and they were excluded.

The National Patient Registry covers all admissions to Swedish hospitals since 1987, [10] and the Cause of Death Registry covers all deaths of Swedish citizens. Records were linked using each person’s unique personal identification number, which is given to everyone who has their permanent residence in Sweden [11].

### Identification of death and 30-day mortality

Data from the Causes of Death Registry were available until 31 December 2012, which allowed at least 12 months’ follow-up after the date of admission to hospital, which was considered the index date of the injury. Mortality was calculated based on the date of death being within 30 days of the index date (30-day mortality) to include most of the patients who died as a direct result of the injury, and to exclude those who died mainly of other causes [12–14].

### Severity of injury

The Injury Severity Score (ISS) has long been regarded as the standard measure of injury severity. In 1996 Osler et al. developed a score based on ICD-9 hospital discharge diagnoses (ICISS), so that they could use big databases with diagnostic codes that had been recorded for administrative purposes [5]. Later studies showed that ICISS calculated from ICD10 was superior [15, 16] and allowed a more accurate estimate of severity of injury [3]. Since then ICISS has been used and evaluated several times both at the European level and in Sweden. [8, 17–22]. A recent meta-analysis also supports the value of this methodology to assess trauma mortality outcome [23].

The Diagnosis-specific Survival Probability (DSP in the formula below) is the proportion of patients with a specific injury code who survived 30 days after the first admission. We omitted duplicate ICD10 codes from the National Patient Registry before we calculated the survival probability. The DSPs were estimated from all injured patients whose details had been primarily extracted from our trauma/injury database, using the main injury diagnosis codes and up to nine secondary codes. The ICISS for individual cases were calculated as the product of DSPs corresponding to the patient’s injury codes (that is, the product of each probability for survival after the injury).

$$ \mathrm{ICISS}={\mathrm{DSP}}_{\mathrm{main}\ \mathrm{diagnosis}}\times {\mathrm{DSP}}_{\mathrm{secondary}\ \mathrm{diagnosis}\ 1}\times {\mathrm{DSP}}_{\mathrm{secondary}\ \mathrm{diagnosis}\ 2}\times {\mathrm{DSP}}_{\mathrm{secondary}\ \mathrm{diagnosis}\dots } $$

### Comorbidity

The Charlson Comorbidity Index (CCI) was calculated using the scale in the original article as described [24] and the ICD-codes from Christensen et al. [25].

### Statistical modelling

To adjust mortality for the severity of injury, the ICISS was used in logistic regression models of 30-day mortality. In the second step we added the sex and age groups to improve the predictive value of the model [17] and to adjust for confounding [26]. A third step included the CCI [24] in the regression model, and as a final step we added calendar year as a covariate in the model.

### Statistical analysis

Incidence/100000 person-years was calculated using national data retrieved from the Statistics Sweden open database for population [27]. The coefficient of variation for estimates of incidence over time was calculated as the ratio of the SD to the mean. Linear regression was used to estimate the trend in incidence of injuries over time. All the models used logistic regression for 30-day mortality.

The discrimination is the model’s ability to separate those who died from those who survived, which was measured by calculation of the area under the receiver operating characteristic curve (AUC) using the C statistic.

Because ICISS may not have a linear relation with the logit of mortality, [28] ICISS and year were modelled both as a linear effect and as a restricted cubic spline in the logistic regression models.

We used the statistics software Stata (StataCorp LP. 2011–15. Stata version 12–14. College Station, TX, USA) for data management and statistical analyses. Probabilities of less than 0.05 were accepted as significant.

## Results

### Patient characteristics

The final study population consisted of 815,846 hospital admissions for the three causes of injury. The age span ranged from 0 to 111 years, mean (SD) age 58 (29) years. “Fall” was the largest group and “assaults” the smallest. More women than men presented with a fall, whereas in the traffic incident and assault groups there were more men. There is a predominance of older people in fall-related injuries whereas the younger ones were more likely to sustain traffic-incident-related and assault-related injuries (Table 1).

**Table 1:** Characteristics of patients by cause of injury

Variable	Fall	Traffic	Assault
Patients	635,074 (78)	153,708 (19)	27,064 (3)
Male	259,759 (41)	93,186 (61)	20,866 (77)
Age in years, mean (SD)	64 (27)	37 (22)	33 (15)
Age			
0–14	70,162 (11)	23,966 (16)	1007 (4)
15–25	27,513 (4)	38,259 (25)	10,480 (39)
26–35	17,937 (3)	19,627 (13)	5360 (20)
36–45	25,356 (4)	19,197 (12)	4425 (16)
46–55	38,657 (6)	17,295 (11)	3403 (13)
56–65	61,672 (10)	14,557 (9)	1540 (6)
66–75	82,697 (13)	9805 (6)	527 (2)
76 and over	311,080 (49)	11,002 (7)	322 (1)

Data are number (%) unless otherwise stated

### Incidence

**Fig. 2:**
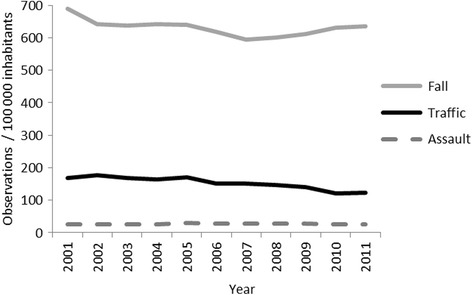
The incidence of injuries /100000 person years over the period by mechanism of injury

The crude numbers of patients/year remained almost constant during the study period, the biggest variation being in the “traffic incidents” group (coefficient of variation 11%). Figure 2 shows the incidence of injury throughout the period. Linear regression analyses showed that fall-related injuries decreased from 689 to 636 hospital admissions / 100,000 inhabitants (*p* = 0.047, annual mean decrease of − 4.71, 95% confidence interval (CI) -9.93 to 0.06), and traffic-incident-related ones from 169 to 123 observations/100000 inhabitants, (*p* < 0.0001, annual mean decrease of − 5.37, 95% CI -6.91 to − 3.82). The incidence of assault-related injuries remained almost unchanged over the period (from 25 to 26 hospital admissions/100000 inhabitants, *p* = 0.45, annual mean increase of 0.11, 95% CI - 0.21 to 0.43). The variability between years probably resulted from the limited number of observations in the assault group.

### Mortality

**Fig. 3:**
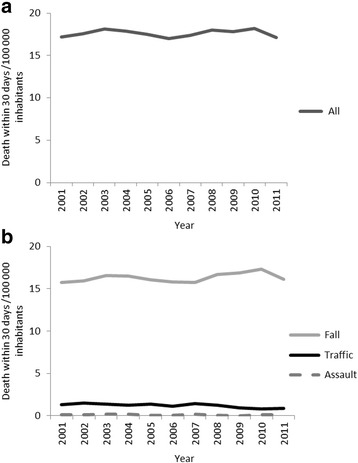
**a**. Crude incidence of death within 30 days (total number of deaths/100000 inhabitants). **b**. Crude incidence of 30-day mortality/100000 inhabitants of injuries over the years by mechanism of injury

The crude overall mortality in the study population remained relatively stable over time (Fig. 3a) (from 17 to 17 dead within 30 days/100000 inhabitants, *p* = 0.72). The crude traffic-related mortality decreased (from 1.3 to 0.9 dead within 30 days/100000 inhabitants, *p* = 0.008, linear regression coefficient − 0.056 see Fig. 3b), as opposed to falls and assaults that remained roughly stable over the study period, although with a large variability in the subgroup with assault injuries.

### Risk-adjusted mortality

Calendar time (year) was not an independent risk factor for mortality within 30 days when adjusted for age, injury severity (ICISS), and CCI (Table 2).

**Table 2:** Logistic regression for death within 30 days after admission (fall, traffic and assault), risk adjusted by ICISS

	OR	*p*	95% CI	95% CI
ICISS	7.20e-06	< 0.001	5.93e-06	8.75e-06
Sex (male as reference)	0.54	< 0.001	0.53	0.56
CCI	1.25	< 0.001	1.24	1.27
Year	1.00	0.008	0.99	1.00
Age, reference is 0–14 years				
15–25	4.04	< 0.001	2.78	5.86
26–35	3.78	< 0.001	2.56	5.60
36–45	3.87	< 0.001	2.63	5.69
46–55	5.89	< 0.001	4.08	8.49
56–65	8.83	< 0.001	6.20	12.58
66–75	20.11	< 0.001	14.21	28.45
76 and older	70.76	< 0.001	50.20	99.76
Fall	1.41	0.001	1.14	1.74
Traffic	0.90	0.340	0.73	1.12
Assault	1.00			

*n* = 815,846

*Abbreviations*: *CCI* Charlson Comorbidity Index, *CI* Confidence Interval, *ICISS* International Classification of disease Injury Severity Score, *OR* Odds Ratio

The model also suggests that the risk for death within 30 days may be higher following fall injury (OR 1.41; 95% CI 1.14 to 1.74), and lower following traffic injury (OR 0.90; 95% CI 0.73 to 1.12), using assault as the reference category.

Modelling death within 30 days for each subgroup suggests an annual decrease of mortality risk for traffic-related injuries (OR 0.95; 95% CI 0.93 to 0.97), and an annual decrease of mortality risk for assault-related injuries (OR 0.93; 95% CI 0.87 to 0.99). The risk for death within 30 days after fall-related injuries did not appear to change over the study period (OR 1.00, 95% CI 0.99 to 1.00). Age and sex were independent risk factors for mortality in fall-related and traffic-incident-related injuries, but not in assaults (Tables 3, 4 and 5).

**Table 3:** Logistic regression for death within 30 days after admission (fall), risk adjusted by ICISS

	OR	*p*	95% CI	95% CI
ICISS	6.06e-06	< 0.001	4.81e-06	7.65e-06
Sex (male as reference)	0.53	< 0.001	0.52	0.55
CCI	1.24	< 0.001	1.23	1.26
Year	1.00	0.195	0.99	1.00
Age, reference is 0–14 years				
15–25	13.42	< 0.001	4.04	44.57
26–35	14.11	< 0.001	4.17	47.71
36–45	15.25	< 0.001	4.65	50.06
46–55	38.08	< 0.001	12.10	119.82
56–65	69.85	< 0.001	22.41	217.67
66–75	167.16	< 0.001	53.82	519.24
76 and older	587.07	< 0.001	189.26	1821.05

*n* = 635 074

*Abbreviations*: *CCI* Charlson Comorbidity Index, *CI* Confidence Interval, *ICISS* International Classification of disease Injury Severity Score, *OR* Odds Ratio

**Table 4:** Logistic regression for death within 30 days after admission (traffic), risk adjusted by ICISS

	OR	*p*	95% CI	95% CI
ICISS	1.27e-05	< 0.001	8.76e-06	.0000183
Sex (male as reference)	0.69	< 0.001	0.60	0.79
CCI	1.22	< 0.001	1.11	1.33
Year	0.95	< 0.001	0.93	0.97
Age, reference is 0–14 years				
15–25	1.69	0.012	1.13	2.56
26–35	1.56	0.049	1.00	2.41
36–45	1.81	0.007	1.17	2.80
46–55	2.37	< 0.001	1.55	3.63
56–65	2.88	< 0.001	1.89	4.40
66–75	5.26	< 0.001	3.47	7.97
76 and older	14.47	< 0.001	9.78	21.41
ICISS	1.64e-05	< 0.001	4.97e-06	.0000544

*n* = 153 708

*Abbreviations*: *CCI* Charlson Comorbidity Index, *CI* Confidence Interval, *ICISS* International Classification of disease Injury Severity Score, *OR* Odds Ratio

**Table 5:** Logistic regression for death within 30 days after admission (assault), risk adjusted by ICISS

	OR	*p*	95% CI	95% CI
ICISS	1.64e-05	<0.001	4.97e-06	.0000544
Sex (male as reference)	0.66	0.129	0.39	1.13
CCI	1.46	0.022	1.06	2.02
Year	0.93	0.022	0.87	0.99
Age, reference is 0–14 years				
15–25	0.75	0.695	0.17	3.24
26–35	1.07	0.929	0.24	4.71
36–45	1.08	0.921	0.24	4.77
46–55	2.02	0.347	0.47	8.80
56–65	2.92	0.161	0.65	13.10
66–75	2.64	0.253	0.50	14.00
76 and older	12.33	0.001	2.67	57.01

*n* = 27,064

*Abbreviations*: *CCI* Charlson Comorbidity Index, *CI* Confidence Interval, *ICISS* International Classification of disease Injury Severity Score, *OR* Odds Ratio

In sensitivity analyses ICISS and year were also modelled as restricted cubic splines in the logistic regression models with similar results (data not shown).

The discriminative value (C-statistics, AUC) of the logistic regression model for death within 30-days including age, sex, year, ICISS and CCI was 0.876 when applied to the entire study population. Subgroup analyses showed an AUC of 0.858 among the fall-related injuries, an AUC of 0.923 among traffic-incident-related injuries, and an AUC of 0.875 among the assault-related injuries.

## Discussion

Using nation-wide health-care registries we identified patients admitted to hospital with injuries caused by falls, traffic incidents, or assaults during an 11-year period. The incidence of fall- and traffic-related injuries decreased over the study period, but there were no notable change in the incidence of assault-related injuries. There was a decrease in mortality risk following traffic-related and assault-related injuries over time, independent of injury severity and baseline patient characteristics such as comorbidity. This may suggest that there have been improvements in prehospital and/or in-hospital management of these injuries. No decrease was, however, seen in the mortality risk following fall-related injuries. These injuries are mainly due to low-energy trauma and may not benefit from management strategies developed for high-energy trauma.

### Strengths of the study

The population-based design with reliable follow-up based on the exact person-based linkage of hospital discharge records with cause of death data, and accurate estimates of the injury severity are notable strengths of this study. The ICISS provides an accurate estimation of the severity of injury [3]. The quality of underlying coding of injuries in the Swedish National Patient Registry has been validated previously, and is accurate to the fourth position of the code [29].

### Limitations of the study

One limitation of the study is that some types of injury are rare, even in a nationwide study, which adds uncertainty to estimates of the severity of injury. A previous study showed that probabilities of diagnosis-specific survival are to a large extent comparable between data from Sweden and the USA [17]. A comparison among eight countries also suggested substantial similarities between countries in terms of ICISS [17]. Another limitation is that the data is collected from a high income country and may not be applicable to middle- or low-income countries The trends in risk-adjusted mortality may reflect changes in the quality of care, but such an interpretation must be cautious, and it is not possible to identify from this study which specific components of care may be of importance. Since the study does not include pre-hospital deaths the design may be less well suited to detect impact of pre-hospital interventions [30, 31]. Further limitations include that this dataset has not been calibrated, but when using the ICISS-method it is rarely done. Finally one could imagine that patients readmitted and deceased after our 30-day limit would change the dataset, however when doing calculations for 90-day mortality (data not shown) the differences were very small. As with all registry data some caution needs to be made regarding data input quality. This issue has been discussed previously for data from the National Swedish Registry [10].

### Strengths, weaknesses, and important differences in results compared with other studies

It has previously been shown that Sweden compares well with other countries in the recording and outcome of injury [17]. Even though coding-errors in ICD-10 are common, the consequences for estimates of the severity of injury were minor in most cases [29].

An increase in the incidence of fall-related, and a decrease in that of traffic-incident-related injuries has previously been reported from the USA [2, 32]. The countries differ in incidence as Sweden has roughly twice the incidence of falls, and a tenth the incidence of traffic-incident-related injuries than the USA [2].

In addition, the incidence of fall-related injuries is difficult to compare with those of other studies because of different inclusion criteria. Most previous studies have focused on a small group, such as those who present to emergency departments, older people, or those with specific fractures, [33–35] whereas this population-based study has included all fall-related admissions to hospital. Crude mortality after fall-related injuries in Sweden is up to twice that in the USA [2]. Some studies have reported no change over time, [32] while we found a small decrease in incidence. This finding is however uncertain due to the limited precision in this estimation.

The incidence of traffic-incident-related injuries estimated in this study is not limited to motor vehicle traffic as it is in the National Trauma Data Bank (NTDB) in the USA, [2] but it includes all traffic-related injuries. Nevertheless our data indicate a mortality that is roughly one tenth of that reported by the NTDB [2]. This difference must be interpreted with caution, because the effect of selection bias among NTDB data is not known but previously published articles has included both pre- and in-hospital death (no exclusion date in mortality stated). Despite the difference in absolute incidence, the focus in our analyses is on the change in mortality over time. After adjusting for other risk factors such as injury severity and comorbidity, there was still a decrease in mortality among the traffic-related injuries. A possible explanation may be an improvement in prehospital or in-hospital care, but this remains largely speculative.

In assault-related injuries, most previous studies have focused on specific mechanisms such as firearms, [36, 37] but because of the low incidence of such injuries in Sweden it is difficult to compare specific mechanisms of injury with other countries [2, 38]. As in the other groups, the decrease in risk-adjusted mortality in this group may also be related to improvements in pre-hospital or in-hospital care but may also reflect residual confounding from trends in specific injury mechanisms within the broad category of assault injuries still unadjusted for.

Although this study was not designed to evaluate changes in medical treatment, improvement in pre-hospital or in-hospital remain a possible explanation for the remaining reduction in mortality over time seen after adjusting for age, sex, injury severity, and comorbidities.

### Meaning of the study: Possible explanations and implications for clinicians and policymakers

This study has indicated a decrease in mortality risk for traffic-, and assault-related injuries over time, independent of age, sex, injury severity, and comorbidities. One potential interpretation is that this reflects improvements over time in prehospital or in-hospital care, or both. No such improvement is seen for fall-related injuries.

### Unanswered questions and future research

The independent contribution of different preventive measures and improvements in interventions in health-care require further study. In our analyses, sex was a significant risk factor for mortality in most groups. While the evaluation of specific health-care interventions [39] should be studied, it is also important to try to understand the mechanism behind the observed association between sex and mortality after injury, because that association was independent of both severity and mechanism of injury. Comorbidity was another independent risk factor and that needs to be further investigated.

## Conclusion

In this population-based study over an 11-year period the incidence of fall- and traffic related-injuries decreased. Risk-adjusted 30-day mortality risk after traffic- and assault-related injuries decreased over time while mortality following fall-related injury remained unchanged over time. This may suggest improved performance of health care interventions mainly targeting high-energy trauma such as traffic- and assault-related injuries.

## Abbreviations

AUC: Area Under the Curve
CCI: Charlson Comorbidity Index
CI: Confidence Interval
DSP: Diagnosis-specific Survival Probability
ICD-9/10: International Classification of Disease version 9/10
ICISS: International Classification of disease Injury Severity Score
ISS: Injury Severity Score
NTDB: National Trauma Data Base
OR: Odds Ratio
SD: Standard Deviation
